# Estimation of Daily Energy Expenditure in Pregnant and Non-Pregnant Women Using a Wrist-Worn Tri-Axial Accelerometer

**DOI:** 10.1371/journal.pone.0022922

**Published:** 2011-07-29

**Authors:** Vincent T. van Hees, Frida Renström, Antony Wright, Anna Gradmark, Michael Catt, Kong Y. Chen, Marie Löf, Les Bluck, Jeremy Pomeroy, Nicholas J. Wareham, Ulf Ekelund, Søren Brage, Paul W. Franks

**Affiliations:** 1 Medical Research Council Epidemiology Unit, Institute of Metabolic Science, Cambridge, United Kingdom; 2 Genetic Epidemiology and Clinical Research Group, Section for Medicine, Department of Public Health and Clinical Medicine, Umeå University Hospital, Umeå, Sweden; 3 Genetic and Molecular Epidemiology Unit, Department of Clinical Sciences, Lund University, Malmö, Sweden; 4 Medical Research Council Human Nutrition Research, Cambridge, United Kingdom; 5 Institute for Ageing and Health, Newcastle University, Newcastle, United Kingdom; 6 Clinical Endocrinology Branch, National Institute of Diabetes and Digestive and Kidney Diseases, National Institutes of Health, Bethesda, Maryland, United States of America; 7 Division of Nutrition, Department of Clinical and Experimental Medicine, Linköping University, Linköping, Sweden; 8 Phoenix Epidemiology and Clinical Research Branch, National Institute of Diabetes and Digestive, and Kidney Diseases, National Institutes of Health, Phoenix, Arizona, United States of America; University of Granada, Spain

## Abstract

**Background:**

Few studies have compared the validity of objective measures of physical activity energy expenditure (PAEE) in pregnant and non-pregnant women. PAEE is commonly estimated with accelerometers attached to the hip or waist, but little is known about the validity and participant acceptability of wrist attachment. The objectives of the current study were to assess the validity of a simple summary measure derived from a wrist-worn accelerometer (GENEA, Unilever Discover, UK) to estimate PAEE in pregnant and non-pregnant women, and to evaluate participant acceptability.

**Methods:**

Non-pregnant (N = 73) and pregnant (N = 35) Swedish women (aged 20–35 yrs) wore the accelerometer on their wrist for 10 days during which total energy expenditure (TEE) was assessed using doubly-labelled water. PAEE was calculated as 0.9×TEE-REE. British participants (N = 99; aged 22–65 yrs) wore accelerometers on their non-dominant wrist and hip for seven days and were asked to score the acceptability of monitor placement (scored 1 [least] through 10 [most] acceptable).

**Results:**

There was no significant correlation between body weight and PAEE. In non-pregnant women, acceleration explained 24% of the variation in PAEE, which decreased to 19% in leave-one-out cross-validation. In pregnant women, acceleration explained 11% of the variation in PAEE, which was not significant in leave-one-out cross-validation. Median (IQR) acceptability of wrist and hip placement was 9(8–10) and 9(7–10), respectively; there was a within-individual difference of 0.47 (p<.001).

**Conclusions:**

A simple summary measure derived from a wrist-worn tri-axial accelerometer adds significantly to the prediction of energy expenditure in non-pregnant women and is scored acceptable by participants.

## Introduction

Glucose metabolism is closely linked to physical activity [1] and maternal glucose homeostasis during pregnancy plays an important role in foetal programming of the endocrine pancreas [2]. However, it is unclear whether higher levels of physical activity during pregnancy are associated with maternal glucose [3]–[5], which is partly because the accurate assessment of physical activity in pregnancy is a major challenge. Perhaps because of this, little is known about suitability of objective methods for physical activity assessment which can be used in large-scale studies of pregnant populations [3].

Accelerometers are becoming increasingly popular in epidemiological studies, and because they are relatively non-invasive, they may be suitable for use in a range of settings, including pregnancy. Moreover, accelerometers may provide a more accurate estimation of physical activity-related energy expenditure (PAEE) than can be obtained from more traditional epidemiological approaches, such as questionnaire-based estimations [6]–[10]. It is however important that the method used is acceptable to the study participant to minimize selection bias and maximize the amount of data obtained; thus studies that seek to evaluate validity and acceptability of monitors are important.

Accelerometers are most commonly attached to the waist using an elastic belt, which, for reasons of discomfort, requires that participants remove the monitor during water-based activities and, generally, also during sleep. This may affect the incompleteness of the data beyond the intended periods of non-wear time because participants forget to re-attach the monitor. Small, waterproof accelerometers allow attachment to the wrist without the need to intermittently remove the monitor, which may improve data completeness. However, accelerometers attached to the wrist may not capture the main body movements that contribute to PAEE as suggested in early laboratory studies comparing uniaxial accelerometry obtained at wrist and waist level [11], [12]. However, a recent study reported similar correlations (r>0.8) for triaxial wrist and waist acceleration with energy expenditure during a laboratory simulation of daily activities [13].

The purpose of this study was to examine whether a simple summary measure of tri-axial acceleration from a wrist-worn accelerometer can contribute to estimated PAEE in pregnant and in non-pregnant women. Further, we compared participant acceptability of accelerometers attached to wrist and hip in a different sample, comprising both men and women.

## Methods

### Ethics statement

Ethical approval was obtained from the Regional Ethical Review Board in Umeå, Sweden, and from the Cambridgeshire research ethics committee, Cambridge, UK.

### Participants

One hundred and eight healthy women (aged 20–35 yrs) residing in the eastern area of the county of Västerbotten, Sweden were recruited through local media advertising and with the assistance of midwives working in local antenatal clinics. Thirty-five of these women were studied during weeks 28–32 of pregnancy, whereas the remainder were non-pregnant. Non-pregnant and pregnant women were of a similar socio-demographic background. The vast majority of non-pregnant women had maintained a stable relationship for at least a year prior to recruitment and reported that they anticipated becoming pregnant in the future. The outcome of the 35 pregnancies (all occurring after the current investigation) was 34 successful deliveries at term (31 vaginal deliveries, 1 planned and 2 acute caesarean sections) and 1 premature birth (including offspring health complications).

Ninety-nine healthy women and men (aged 22–65 yrs) from Cambridgeshire in the UK were recruited through local media advertising to a study aimed at evaluating the validity of a physical fitness test.

Participants in both studies were initially interviewed by telephone and asked to complete a modified version of the Rose Angina Questionnaire to identify major contraindications to exercise [14]. Exclusion criteria included recent major cardiovascular events, recent physically debilitating surgical procedures, serious unmanaged psychiatric disorders, illicit drug dependency, and an inability to commit fully to the study procedures. The objectives and procedures of the study were explained in detail to the participants, after which they provided written and verbal informed consent.

### Study design

In the Swedish study centre, the accelerometer was worn on the wrist continuously for 10 days of free-living and total energy expenditure (TEE) was simultaneously measured using the doubly labelled water (DLW) method. Additionally, resting energy expenditure (REE) and anthropometric characteristics were measured [15].

In the British study centre, participants were asked to wear one hip and one wrist accelerometer for 7 days of free-living. At the end of the monitoring period, participants were asked to indicate the acceptability of each monitor on a 10 level ordinal rating scale, where 1 represented a very low level of acceptability and 10 represented a very high level of acceptability.

### Accelerometer

The accelerometer (GENEA, Unilever Discover, Sharnbrook Bedfordshire, UK) comprised a tri-axial STMicroelectronics accelerometer (LIS3LV02DL) with a dynamic range of ±6 g (1 g = 9.81 m·s^−2^), as described elsewhere [13]. The acceleration was sampled at 40 Hz (Sweden) and 80 Hz (UK) and data were stored in g units for offline analyses. The accelerometer (12×29×37 mm) was attached to the wrist with a nylon weave strap (both groups) and to the hip with an elastic belt (UK sample only). In order to ensure an even balance of left and right wrist positioning in the Swedish study, every second participant entering the study was asked to wear the accelerometer on the left wrist and every other to wear it on their right wrist, irrespective of hand dominancy. In the British study, both accelerometers were worn on the non-dominant body side. Participants were instructed to wear the accelerometer on the wrist continuously but to remove the hip-placed accelerometer during water-based activities and sleeping. A diary was provided to assess monitor non-wear time.

### Signal processing

Accelerometer non-wear time was estimated on the basis of the standard deviation and the value range of each accelerometer axis, calculated for consecutive blocks of 30 minutes. A block was classified as non-wear time if the standard deviation was less than 3.0 mg (1 mg = 0.00981 m·s^−2^) for at least two out of the three axes or if the value range, for at least two out of three axes, was less than 50 mg. Thresholds were based on lab experiments in which thirty GENEA accelerometers were left motionless on a flat, stable surface for 30 minutes, showing that the standard deviation of an acceleration signal (which has some inherent noise) is 2.6 mg during non-motion. Therefore, the 3.0 mg threshold will allow a maximal increase of 0.4 mg in the standard deviation, which when expressed in angular orientation of the acceleration sensor corresponds to a standard deviation of 1.6 degrees [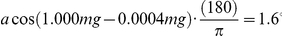]. Phan et al. showed that the acceleration of the chest in a resting person resulting from the breathing movement alone has an amplitude of 10 mg, while the vibrations resulting from the heart beat have a peak-to-peak amplitude of 80 mg [16]. Therefore, even the tiniest wrist movements are likely to be picked up by the method as described above.

Participants for whom more than 50% of the wrist data was classified as non-wear were excluded from further analyses (two pregnant women from the Swedish study). For the remainder of the participants, non-wear time segments were labelled as missing.

Next, a simple summary measure was derived from the raw acceleration signals, involving a filtering stage to extract the accelerations related to body movement using a fourth-order Butterworth band pass filter (ω_0_: 0.2–15 Hz), followed by the calculation of the vector magnitude (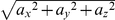). The resulting signal was then averaged over intervals of one second. Three basic approaches for the imputation of movement during non-wear segments were evaluated: i) no imputation, with non-wear time regarded as no movement (Acc_0_); ii) imputation of non-wear time by the average movement during wear time for that participant (Acc_1_); and iii) imputation of non-wear time using the available wear time data at similar times on other days for each participant (Acc_2_). Here, Acc_2_ is assumed to be best capable of dealing with large periods of missing data as it takes into account the 24-hour cycle of human behaviour. Finally, the average was calculated for each participant. All signal processing was done in R (http://cran.r-project.org) using package Signal.

### Anthropometry and energy expenditure (Swedish study only)

Participants visited the Clinical Research Center at Umeå University Hospital the morning after a 10-hour fast on day 1 of the study. Height was measured using a wall-mounted stadiometer to the nearest 0.5 cm, body weight was measured using a calibrated digital scale (Tanita Corporation, Tokyo, Japan) to the nearest 0.1 kg, and arm length, defined as the distance between the lateral edge of the acromion and the processes styloidius of the ulna, was measured using a non-stretchable measurement tape to the nearest 0.5 cm. Arm length was included as it may explain variation in wrist acceleration, because of the mechanical relationship between radius (arm length) and acceleration of a rotating object (accelerometer). REE was measured under thermoneutral conditions for 30 minutes using indirect calorimetry with a ventilated hood (Deltatrac II, Datex-Ohmeda, Inc., WI, USA) placed over participants who were lying still and quietly on a bed without sleeping for 30 minutes. The accelerometer was thereafter fitted to the wrist. Prior to the completion of the testing session, each participant was given an accurately weighed oral dose of DLW stable isotopes (0.07 g ^2^H_2_O and 0.174 g H_2_^18^O per kg body weight). In addition to a pre-dose urine sample, 10 additional urine samples were collected, one for each of the 10 days that followed the day of dosing. The time of each sample was noted by the participant in a log. Urine samples were stored in plastic urine vials at +4°C until collection by the research team. Samples were subsequently frozen at −20°C pending analysis. Isotopic enrichments of dose and urine samples were analyzed at MRC Human Nutrition Research, Cambridge, UK [17]. PAEE was calculated as 0.9× TEE – REE [17].

### Statistics

Linear regression analysis was applied to develop prediction models for PAEE. A priori, separate models for pregnant and non-pregnant women were evaluated, including the following variables: wrist acceleration, body weight, age, body height, body side (dominant wrist vs. non-dominant wrist), and arm length. To determine whether the relationship between wrist acceleration and PAEE was affected by body side (dominant or non-dominant wrist attachment) an interaction analysis was performed using a multiplicative term (acceleration×body side) plus the marginal effects for this term. Finally, a leave-one-out cross validation analysis was applied to estimate model performance outside the training dataset [18], which was evaluated by modified Bland-Altman plots using criterion PAEE on the horizontal axis [19]. All models are expressed in MJ day^−1^ as this is regarded to be the most appropriate way to evaluate the contribution of wrist acceleration independent of body weight to the total explained variation in PAEE (MJ day^−1^) from a statistical point of view [9]. However, we acknowledge the preference for other normalisation approaches from a biological perspective [20], so regression models for PAEE expressed in Joules min^−1^ kg^−1^ are reported in the appendix S1 (**table A**) to facilitate a more complimentary interpretation of our results.

Acceptability scores (UK study) for wrist and hip accelerometer placements are summarised as median (inter-quartile range) for wrist and hip attachments and stratified by sex. The equality in acceptability between wrist and hip sites was tested using a two-sided Wilcoxon paired signed-rank test and a non-paired signed-rank test for male vs. female comparisons.

All analyses were carried out in the open-source tool R (http://www.r-project.org). An alpha level of p<.05 was regarded as statistically significant.

## Results

Participant characteristics are shown in **table 1**. In the Swedish study no significant differences were found between pregnant and non-pregnant women in their PAEE, REE, TEE, physical activity level (PAL = TEE/REE), and wrist acceleration (see table 1).

**Table 1:** Participant characteristics providing at least one valid day of accelerometer data.

	Non-pregnant women	Pregnant women	Non-pregnant women	Men
	(SWE)	(SWE)	(UK)	(UK)
N	65	30	46	37
Body mass (kg)	77.3±19.2	76.9±14.8	63.2±9.7	77.5±10.8***
Height (m)	1.67±0.07	1.67±0.06	1.64±0.07	1.77±0.07***
Age (yrs)	28±4	30±3**	41±12	43±14
Arm length (cm)	57±4	59±4**	-	-
BMI (kg·m^−2^)	27.8±6.6	27.7±5.3	23.4±3.3	24.7±3.3
PAEE (MJ/d)	3.55±1.22	3.75±0.91	-	-
TEE (MJ/d)	11.17±1.63	11.17±1.24	-	-
REE (MJ/d)	6.50±0.98	6.30±0.53	-	-
PAL	1.73±0.23	1.78±0.16	-	-
Acc_2_ (g) wrist	0.122±0.019	0.123±0.023	0.123±0.024	0.120±0.029
Acc_2_ (g) hip	-	-	0.065±0.019	0.065±0.021
EWT wrist (%)^†^	99.4 (98.2–99.8)	99.2 (98.1–99.7)	98.5 (88.4–99.4)	99.1 (97.9–100.0)
EWT hip (%)^†^			64.9 (60.4–67.6)	64.6 (61.0–67.3)

[Values are mean ± standard deviation, or ^†^median (Inter-Quartile Range); SWE, Sweden; UK, United Kingdom; t-test to test for differences between pregnant (28–32 weeks of gestation) and non-pregnant women (SWE) or between men and women (UK), where *p<0.05, **p<0.01; PAEE: physical activity-related energy expenditure; TEE: total energy expenditure; REE: resting energy expenditure; PAL: physical activity level calculated as TEE/REE; Acc_2_: average acceleration (g) where non-wear time was imputed by all wear-time data at similar time of the day for that participant; EWT: estimated wearing time].

### Energy expenditure (Swedish study)

**Figure 1:**
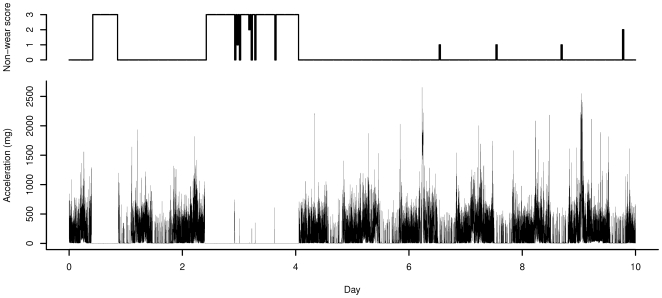
Example of the acceleration (Acc_2_) and the non-wear detection score (number of acceleration sensor axes that meet the threshold for non-wear) for one participant in the Swedish study.

No accelerometer data was retrieved from 9 participants due to failure to wear the monitor or technical errors. Three of these participants also failed to collect urine samples, resulting in 99 participants with a complete set of data. Owing to suboptimal power management in the monitor firmware (a problem that appears to affect measurement duration in this version of the GENEA monitor), the data collection period was curtailed for some participants in the Swedish study. On average, 8.2 days of data were collected (median: 9.6, 25^th^ percentile: 7.6 days). In 97 out of these 99 participants, at least one day worth of data was collected. These figures relate to the length of the raw data and may partially represent periods of non-wear. According to the diaries, all participants wore the wrist accelerometers for the entire duration of the 10 day monitoring period. However, the non-wear detection classified two participants out of the 97 as having worn the monitor for less than 50% of the measurement duration; these individuals were thus excluded from further analyses. An example of the acceleration (Acc2) and the non-wear detection over time in one Swedish participant is shown in **Figure 1**. Next, regression analysis was done in two groups separately: i) for all participants in whom more than 7 days of data were collected (N = 74, 26 pregnant and 48 non-pregnant women) and ii) for the first three days of data in all participants for whom one or more days of data was collected (N = 95; 30 pregnant and 65 non-pregnant women).

**Figure 2:**
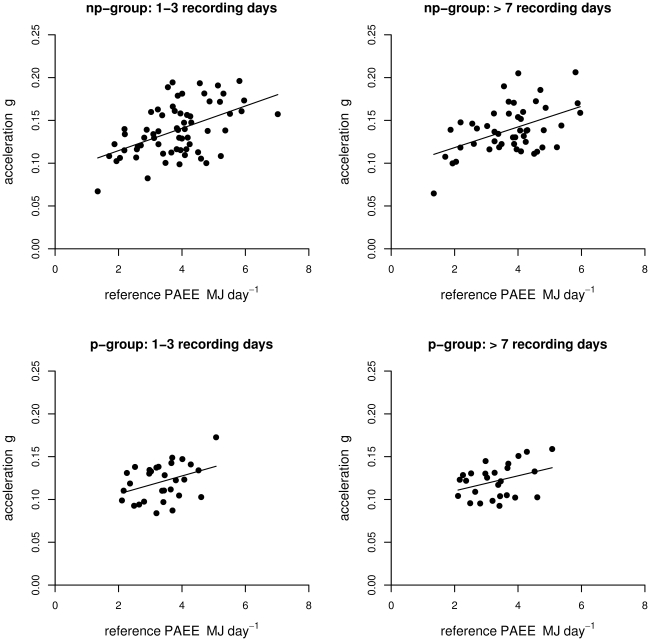
Acceleration (Acc_2_) as a function of physical activity-related energy expenditure (PAEE) in MJ day^−1^ for p-group (pregnant) and np-group (non-pregnant). Lines represent the best fit of the data. See table 3 (model 1) for regression equation.

The average space ratios (Ko/Kd) for non-pregnant and pregnant women were 1.034 and 1.030, respectively. Significant correlations were found between accelerometer output and PAEE (**table 2** **and** **3**). A graphical evaluation of Acc_2_ as a function of PAEE is shown in **figure 2**. In non-pregnant women, Acc_2_ explained 24% (1–3 days of data. SE: 1.00 MJ) and 21% (>7 days of data. SE: 0.98 MJ) of the variation in PAEE. Introducing body weight to the model increased the explained variation for the 1–3 days of data model and the >7 days of data model to 31% (SE: 0.95 MJ) and 31% (SE: 0.92 MJ), respectively (table 2). No significant correlation was found between PAEE and body weight (p = 0.21), nor between PAEE and arm length (p = 0.12) (table 2). Further, no interaction was found between body side and Acc_2_ in non-pregnant women (p = 0.62), (table 3). Acc_2_ and body weight were not significantly correlated to each other in non-pregnant women: r −0.16, p = 0.20 (1–3 day of data), r −0.27, p = 0.07 (>7 day of data). In pregnant women, Acc_2_ over the first three days explained 11% (SE: 0.71 MJ) of the variation in PAEE, and after addition of body side to the model this increased to 27% (SE: 0.65 MJ). None of the models significantly explained variation in PAEE during pregnancy when >7 days of data was used. In pregnant women, there was also no significant correlation between body weight and PAEE (p = 0.95). Acc_2_ and body weight were not significantly correlated to each other in pregnant women: r −0.01, p = 0.94 (1–3 day of data), r −0.17, p = 0.42 (>7days of data). In both pregnant and non-pregnant women, squared wrist acceleration did not significantly contribute to the explained variation by wrist acceleration alone, thus providing support for a linear model analysis of the data (models not shown).

**Table 2:** Overview of regression models for predicting PAEE (MJ day^−1^), stratified by amount of accelerometer information used.

Group	Model input	>7 days			1–3 days			1–3 days, participants with >7 days		
		N	SE	R^2^	N	SE	R^2^	N	SE	R^2^
*Non-*	Acc_0_	48	0.97	0.22***	65	0.99	0.25***	48	0.98	0.21***
*pregnant*	Acc_1_	48	0.99	0.19***	65	1.01	0.22***	48	1.00	0.17***
*women*	Acc_2_	48	0.98	0.21***	65	1.00	0.24***	48	0.98	0.20***
	Weight	48	1.09	0.01 ns	65	1.13	0.03 ns	48	1.09	0.01 ns
	Acc_2_+Weight	48	0.92	0.31***	65	0.95	0.31***	48	0.94	0.27***
	Acc_2_+Body side § (with interaction)	48	0.99	0.18*	65	1.01	0.23*	48	1.00	0.18*
*Pregnant*	Acc_0_	26	0.76	0.08 ns	30	0.73	0.08 ns	26	0.74	0.13*
*women*	Acc_1_	26	0.75	0.09 ns	30	0.72	0.10 ns	26	0.73	0.15*
	Acc_2_	26	0.75	0.09 ns	30	0.71	0.11*	26	0.73	0.15*
	Weight	26	0.81	−0.04 ns	30	0.76	−0.02 ns	26	0.81	−0.04 ns
	Acc_2_+Weight	26	0.77	0.05 ns	30	0.72	0.09 ns	26	0.74	0.12 ns
	Acc_2_+Body side (with interaction)	26	0.71	0.19 ns	30	0.65	0.27*	26	0.66	0.30*

[SE: Residual standard error; ***: p<.001; **: p<.01; *: p<.05; ns: not significant; § no significant contribution from Body side (values based on forced inclusion); Acc: acceleration, where Acc_0_: not based on non-wear detection and succeeding imputation; Acc_1_: average acceleration (g) where non-wear time was imputed by the average of all wear-time data for that participant; Acc_2_: average acceleration (g) where non-wear time was imputed by all wear-time data at similar time of the day for that participant; Body side, dominant wrist vs. non-dominant wrist].

**Table 3:** Regression models for PAEE (MJ day^−1^) in non-pregnant and pregnant Swedish women.

	*Non-pregnant women*			*Pregnant women*		
Model 1	Coefficients	SE	p	Coefficients	SE	p
Constant	1.11	0.73	0.13	1.568	0.946	0.11
	(1.16)	(0.59)	(0.05)	(1.779)	(0.759)	(0.03)
Acc_2_ (g)	18.97	5.13	<.001	14.276	7.692	0.08
	(19.21)	(4.19)	(<.001)	(13.052)	(6.191)	(<0.05)

Model 2	Coefficients	SE	p	Coefficients	SE	p
Constant	−0.873	1.009	0.39	1.26	1.63	0.45
	(−0.429)	(0.808)	(0.60)	(1.22)	(1.04)	(0.25)
Acc_2_ (g)	22.553	5.005	<.001	14.59	7.96	0.08
	(21.014)	(4.040)	(<.001)	(13.13)	(6.23)	(<.05)
Weight (kg)	0.019	0.007	<.05	0.00	0.02	0.82
	(0.017)	(0.006)	(<.01)	(0.01)	(0.01)	(0.43)

Model 3	Coefficients	SE	p	Coefficients	SE	p
Constant	0.76	1.11	0.5	3.856	1.408	<.05
	(0.49)	(0.93)	(0.6)	(3.759)	(1.01)	(<.01)
Acc_2_ (g)	21.33	8.39	<.05	−4.780	12.038	0.71
	(24.75)	(7.05)	(<.001)	(−3.447)	(8.647)	(0.69)
Body side	0.89	1.59	0.58	−4.238	1.886	<.05
	(1.01)	(1.26)	(0.43)	(−4.038)	(1.427)	(<.05)
Acc_2_ (g)×Body side	−5.57	11.24	0.62	33.762	15.485	<.05
	(−8.22)	(9.04)	(0.37)	(32.156)	(11.693)	(<.05)

[Values are based on all participants with >7 days of data; numbers in brackets are results from the first three days in all participants that had at least one day of data; Body side: 1 = accelerometer attached to dominant wrist; 0 = accelerometer attached to non-dominant wrist; SE: Residual standard error; Acc_2_: average acceleration (g) where non-wear time was imputed by all wear-time data at similar time of the day for that participant].

**Figure 3:**
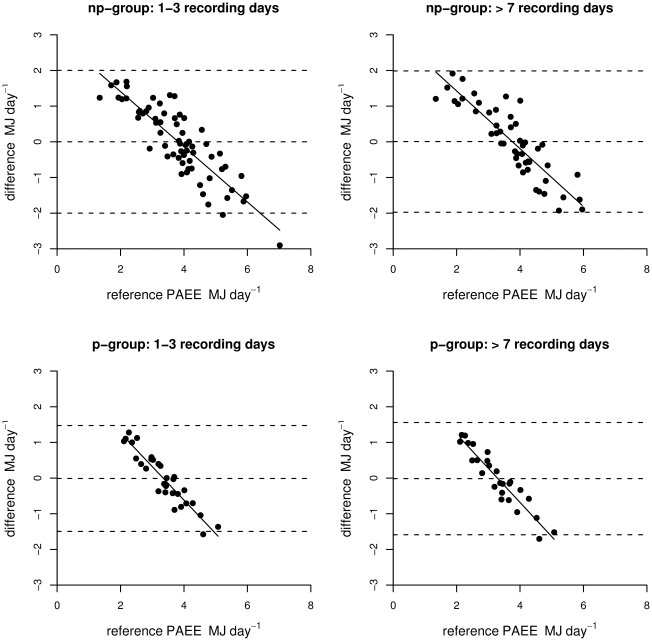
Error of estimated physical activity-related energy expenditure (PAEE) in MJ day^−1^ based on acceleration (Acc_2_) as a function of reference PAEE for p-group (pregnant) and np-group (non-pregnant). Dashed lines represent the average difference and 95% confidence intervals (−2.00–2.00 np-group 1–3 days; −1.97–1.99 np-group >7days; −1.50–1.47 p-group 1–3 days; −1.59–1.56 p-group >7 days).

In the cross-validation based on the leave-one-out approach, PAEE estimates based on Acc_2_ remained significantly correlated to measured PAEE in non-pregnant women, but this correlation was not statistically significant in pregnant women (**table 4**). In pregnant women, the interaction between body side and Acc_2_ was weaker in the cross-validation analysis although still statistically significant in the shorter measurement period and larger sample size (see table 4). A graphical evaluation (modified Bland & Altman plot) of the PAEE estimation error against measured PAEE (cross-validation) is shown in **figure 3** for models based on Acc_2_ and body weight (non-pregnant women) and Acc_2_ with body side (pregnant women).

**Table 4:** Cross-validation using the leave-one-out method of regression models in which PAEE (MJ day^−1^) is the dependent variable.

		>7 days				1–3 days			
Group	Model input	N	RMSE	Bias	R^2^	N	RMSE	Bias	R^2^
*Non-pregnant women*	Acc_2_	48	1.01	−0.01 ns	0.14**	65	1.01	0.00 ns	0.19***
	Acc_2_+Weight	48	0.95	−0.01 ns	0.23***	65	1.03	−0.01 ns	0.26***
*Pregnant women*	Acc_2_	26	0.79	0.02 ns	−0.03 ns	30	0.74	0.01 ns	−0.01 ns
	Acc_2_+Body side (with interaction)	26	0.78	0.02 ns	0.04 ns	30	0.68	0.01 ns	0.15*

[Acc_2_ average acceleration (g) where non-wear time was imputed by all wear-time data at similar time of the day for that participant; RMSE: Root mean square of the error; body side, monitor attachment to dominant wrist vs. non-dominant wrist;

***: p<.001;

**: p<.01;

*: p<.05;

ns: not significant].

### Participant acceptability (UK study)

**Figure 4:**
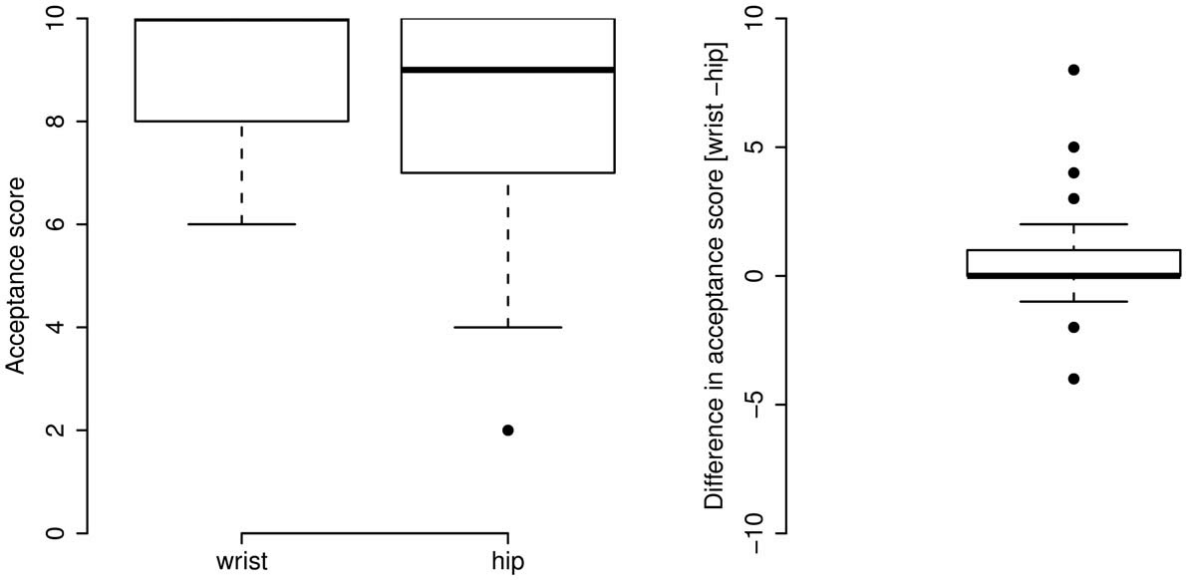
Box plot of acceptability scores for hip and wrist attachment of accelerometer (left panel) and box plot of the within-individual difference (right panel) for UK sample.

Fourteen UK participants were excluded, as acceptability scores were not collected, and two additional participants were excluded, as no acceleration was recorded for either the wrist or the hip, resulting in 83 participants with data available for analysis. The acceptability score for wrist attachment was 0.47 higher than hip attachment (p<.001). The median (IQR) score for wrist attachment was 9 (8–10), and the corresponding value for hip attachment was 9 (7–10) as shown in **figure 4**. When stratified by sex, men scored the wrist placement on average 0.51 higher (IQR: 0–1) compared to the hip placement (p = 0.02). Women scored the wrist placement 0.43 higher (IQR: 0–1), a difference which was not statistically significant. No significant difference was found between sexes for the wrist scores (p = 0.57) or for the hip scores (p = 0.89). The estimated percentage of wear time was higher for wrist attachment (median: 99%) compared with hip attachment (median: 65%) in the UK sample (t = −14.6, p<.001). Sixty five percent wear time represents 97.5% of waking hours (assuming 16 waking hours per day). Wrist acceleration explained 45.3% of the variation in hip acceleration (p<.001), which was not improved by addition of BMI (p = 0.68), age (p = 0.07), or body weight (p = 0.68) to the regression model.

## Discussion

The results from the present study suggest that a simple summary measure derived from a tri-axial accelerometer attached to the wrist contributes significantly to the explained variation in daily PAEE in non-pregnant women but we did not find strong associations in pregnant women. The difficulty in assessing PAEE in pregnant women may be related to the unique patterns of movement and energy metabolism resulting in reduced variance in PAEE (0.91 MJ vs. 1.22 MJ in non-pregnant women). There are no indications that the accuracy of the assessment of TEE is compromised in pregnant women. Although some have argued that the increase in body-weight during pregnancy to some extent will be associated with the binding of ^2^H in newly synthesized protein and fat and with a gradual increase in the body water pool, each of these effects has been estimated to account for <1% mean bias [21]. The day-to-day variability in basal metabolic rate and energy expenditure due to the synthesis of protein and fat in new tissue in pregnancy has not been evaluated. It is possible that an increased day-to-day variability of these energy components may have biased the estimation of average BMR and PAEE over the 10-day measurement period within pregnant women, but we are unaware of any studies which have evaluated this possibility.

The side of the body to which the monitors were attached, but not arm length, significantly contributed to the explained variation in PAEE in pregnant women but not in non-pregnant women; to our knowledge, this observation has not been reported elsewhere, and there is no clear physiological or biomechanical explanation for this. One possible explanation may be that pregnant women frequently caress their belly, an activity which may not increase PAEE appreciably but significantly increase average wrist acceleration.

Body weight did not correlate with PAEE in this sample of women. Several other studies have shown significant correlations between body weight and PAEE in non-pregnant women [22], [23]. The absence of an association between body weight and PAEE reported here may be explained by the lower variation in PAEE in the current study compared with previous studies [22], [23].

Accelerometer output is usually expressed in manufacturer-dependent output values (i.e. “counts”). A count is an arbitrary unit aimed to be proportional to the average acceleration in a specified period of time (epoch) [24]. Counts are difficult to interpret as the underlying data processing method and assumptions are often concealed from the end-user. Further, valuable information is often lost in the process of computing the count, e.g. the frequency content. Unlike many traditional accelerometers, the output from raw accelerometers, such as the type used in this study, is not summarized during data collection (by the monitor), thus providing the end-user with greater control over data processing. Therefore, raw accelerometry (e.g., movement data expressed in g) facilitates easier interpretation and may be of particular value for studies which aim to compare data collected from different monitors but it has so far not been used in large studies. Accelerometer devices, based on inertial or seismic acceleration sensors (MEMS), which allow for raw data storage at a relatively high sampling frequency, have been previously used in gait analysis [25], [26] and ambulant activity classification [27], [28]. Since raw accelerometer data can be re-processed, the re-evaluation of such data at a future date, when more advanced signal processing approaches become available, is possible. For example, differentiating activity types has previously been shown to improve the accuracy of energy expenditure estimation from waist accelerometry and may also be advantageous for wrist accelerometry [29], [30]. To this end, it is likely that vector magnitude of wrist acceleration would not relate very strongly to physiological intensity across biomechanically different activities such as cycling, driving, and walking.

The accuracy of estimated PAEE in the current study may be limited by the simple summary measure derived from the accelerometer data. The metric chosen (band-pass frequency filtering and vector magnitude) was designed to represent the magnitude of body acceleration as a scalar and has been used in previous studies [29], [31]. This fairly rudimentary metric was used because wrist accelerometry has so far not been evaluated against the DLW method. The initial assessment of wrist worn accelerometry should therefore include the evaluation of an easily interpretable metric. Another element of signal processing that may be improved is the non-wear detection, perhaps with evaluation against direct observation.

Owing primarily to monitor power failure (which is unrelated to participant acceptability), the monitoring period was substantially curtailed in a subsection of the dataset. To determine whether this limitation influenced the validity of the data, we conducted sensitivity analyses for either 1–3 days or >7 days of monitoring. The former analysis has the advantage of a larger sample size and may also be more reflective of typical monitoring periods in large epidemiological studies. The smaller sample with >7 days of data may have resulted in weaker correlations, despite overlapping to a greater extent with the criterion measurement period. However, as we show, neither sampling strategy resulted in markedly different conclusions about the validity of the monitor for the assessment of PAEE. However, it should be noted that the derived models lack precision on the individual level, with RMSE values only slightly lower than the between-individual standard deviation in PAEE.

A direct comparison between the accuracy of wrist-mounted and hip-mounted monitors would have been informative, but was not possible due to a limited number of accelerometer devices available at the start of this study. Nonetheless, the current findings in non-pregnant women are comparable to the accuracy reported for accelerometer positioned on the hip or lower back [9], [32]–[34]. For example, triaxial acceleration measured at the lower back added 33% to the explained variance in PAEE (MJ day^−1^) in a study by Plasqui et al. [22] and 16–23% in a follow up study with a different accelerometer by Bonomi et al. [33]. The accelerometer contribution of 24% for non-pregnant women as found in the current study falls in between these values. Further, we observed a relatively high correlation between wrist and hip acceleration in the acceptability study, an association which was not mediated by body weight or BMI. However, it should be noted that comparisons against other studies are limited by differences in study design, study population, monitor type, and data analysis.

Our evaluation of monitor acceptability included both men and women and indicate a high level of acceptability for both placement sites, with wrist or hip accelerometry being tolerated by almost everyone in the sample; on average men tended to favour wrist attachment over hip attachment, while no significant preference was found for women. The estimated percentage of wear time was significantly higher when the monitor was worn on the wrist, which was predominantly a result of monitor-specific wear instructions to the participant. When the acceptability scores are interpreted in the context of the estimated percentage of wear time, the majority of the participants do not seem to object to wearing a wrist accelerometer 24 hrs/day. We assessed acceptability by asking the participants to wear both accelerometer devices at the same time for one week. Wearing the monitors in parallel standardizes possible confounding variables such as participant mood when scoring the monitors, the type of physical activity performed during the wearing period and the type of clothes worn in combination with the accelerometers. However, it should be noted that the acceptability of each device is then evaluated in the context of wearing two devices, which may differ from the acceptability of wearing a single accelerometer. Further, acceptability is likely to be population-dependent and the findings in this sample of healthy adults from the Cambridge area in the UK may therefore not generalise to other populations. Information about monitor acceptability during specific activities in occupational settings, commuting, and leisure time may be important to optimize monitor acceptability and therefore enhance compliance in future studies.

In conclusion, a simple summary measure derived from a non-invasive tri-axial wrist accelerometer contributes significantly to the explained variance in daily PAEE in non-pregnant women, thus demonstrating its utility for large-scale physical activity assessment in women. However, we were unable to differentiate PAEE in pregnancy with this method. Future development of more advanced analytical procedures may further improve the estimation of energy expenditure.

## Supporting Information

Appendix S1 Table A: Models of PAEE (J min^−1^ kg^−1^) as the dependent variable based on all with more then seven days of data; numbers in brackets are results from the first three days in all participants that had at least one day of data.
(DOC)
