# Supplementary material for: Estimation of Daily Energy Expenditure in Pregnant and Non-Pregnant Women Using a Wrist-Worn Tri-Axial Accelerometer
Source: PLoS One. 2011 Jul 29;6(7):e22922. doi: 10.1371/journal.pone.0022922 (PMC3146494; doi:10.1371/journal.pone.0022922)
Supplement: Appendix S1 — Table A: Models of PAEE (J min−1 kg−1) as the dependent variable based on all with more then seven days of data; numbers in brackets are results from the first three days in all participants that had at least one day of data. (DOC) [file pone.0022922.s001.doc]

**Appendix S1**

**Table A:** Models of PAEE (J min^-1^ kg^-1^) as the dependent variable based on all with more then seven days of data; numbers in brackets are results from the first three days in all participants that had at least one day of data.

|  | *Non-pregnant women*  *N = 65 (N = 48)* | | | *Pregnant women*  *N = 30 (N = 26)* | | |
| --- | --- | --- | --- | --- | --- | --- |
| **Independent** | Coefficients | SE | p | Coefficients | SE | p |
| Constant | 0.192 | 7.261 | .98 | 12.287 | 9.926 | .23 |
|  | (6.564) | (6.015) | (.280) | (17.400) | (8.440) | (< .05) |
| Acc_2_ (g) | 248.584 | 51.117 | < .001 | 157.565 | 80.696 | .06 |
|  | (210.743) | (42.516) | (< .001) | (112.892) | (68.879) | (.11) |
| Model | R^2^ = 0.33 | SE: 9.730 | < .001 | R^2^ = 0.10 | SE: 7.900 | .06 |
|  | (0.27) | (10.127) | (< .001) | (0.05) | (7.935) | (.11) |
|  | Leave-one-out cross validation: | | | Leave-one-out cross validation: | | |
|  | R^2^ = 0.26 (0.22); p < .001 (< .001) | | | R^2^ = 0.00 (-0.03); p = .36 (= .67) | | |

[SE: Residual standard error; Acc_2_: acceleration based on the imputation of non-wear time by all existing data at similar times of the day for that participant]
